# Possible Interaction between ZnS Nanoparticles and Phosphonates on Mediterranean Clams *Ruditapes decussatus*

**DOI:** 10.3390/molecules28062460

**Published:** 2023-03-08

**Authors:** Wiem Saidani, Imen Bouzidi, Abdelhafidh Khazri, Samir Ghannem, Iyadh Aouani, Anis Fkiri, Soufiane Touil, Mohammed I. Alghonaim, Sulaiman A. Alsalamah, Ashraf A. Qurtam, Hamouda Beyrem, Fehmi Boufahja, Badreddine Sellami

**Affiliations:** 1 Laboratoire de Biosurveillance de l’Environnement (LBE), Unité d’Ecotoxicologie et d’Ecologie Côtière (GREEC), Faculté des Sciences de Bizerte, Zarzouna-Bizerte 7021, Tunisia; 2 Laboratory of Hetero-Organic Compounds and Nanostructured Materials (LR18ES11), Faculty of Sciences of Bizerte, University of Carthage, Zarzouna 7021, Tunisia; 3 Biology Department, College of Science, Imam Mohammad Ibn Saud Islamic University (IMSIU), Riyadh 11623, Saudi Arabia; 4 National Institute of Marine Sciences and Technologies, Tabarka 8110, Tunisia

**Keywords:** ZnS NPs, hydroxyphosphonates, biomarkers, oxidative stress, *Ruditapes decussatus*

## Abstract

This study aims to evaluate the toxicity of ZnS nanoparticles (ZnS NP50 = 50 µg/L and ZnS NP100 = 100 µg/L) and diethyl (3-cyano-1-hydroxy-2-methyl-1-phenylpropyl)phosphonate or P (P50 = 50 µg/L and P100 = 100 µg/L) in the clams *Ruditapes decussatus* using chemical and biochemical approaches. The results demonstrated that clams accumulate ZnS NPs and other metallic elements following exposure. Moreover, ZnS NPs and P separately lead to ROS overproduction, while a mixture of both contaminants has no effect. In addition, data showed that exposure to P100 resulted in increased levels of oxidative stress enzyme activities catalase (CAT) in the gills and digestive glands. A similar trend was also observed in the digestive glands of clams treated with ZnS100. In contrast, CAT activity was decreased in the gills at the same concentration. Exposure to ZnS100 and P100 separately leads to a decrease in acetylcholinesterase (AChE) levels in both gills and digestive glands. Thus, AChE and CAT after co-exposure to an environmental mixture of nanoparticles (ZnS100) and phosphonate (P100) did not show any differences between treated and non-treated clams. The outcome of this work certifies the use of biomarkers and chemical assay when estimating the effects of phosphonate and nanoparticles as part of an ecotoxicological assessment program. An exceptional focus was given to the interaction between ZnS NPs and P. The antioxidant activity of P has been demonstrated to have an additive effect on metal accumulation and antagonistic agents against oxidative stress in clams treated with ZnS NPs.

## 1. Introduction

Contaminants are unavoidably released into aquatic ecosystems and eventually reach sediment and water as a result of anthropogenic actions [1,2].

Nanoparticles and phosphonates are among the most widely used products in the world [3] and are detected at different concentrations in different compartments of marine ecosystems [4]. These contaminants have harmful effects on aquatic organisms through interference with a range of biological and biochemical pathways [1,2]. Most species can accumulate nanoparticles and phosphonates from contaminated sources, leading to contamination of the entire ecosystem [4] and consequently humans through the food chain [5].

Zinc sulfide nanoparticles (ZnS NPs) have been used for a large number of applications [6]. Because of photochemical processes and dissociation, these types of particles can release toxic metal ions into the aquatic environment [7]. In addition, ZnS NPs have been shown to exhibit some changes in the physicochemical parameters of bodies of water, such as dissolved oxygen level and pH, in turn regulating water quality and hence affecting aquatic fauna [8]. The problem of its toxicity in aquatic species depends on their size, charge, and duration of exposure according to Aye et al. [9], but no data are yet available in marine waters.

Among phosphonates, diethyl (3-cyano-1-hydroxy-2-methyl-1-phenyl propyl) phosphonate (P) is a novel type of hydroxy phosphonate used as a pharmaceutical compound, having received considerable attention for its biological activity as an antibiotic [10] and enzyme inhibitor [11] and for its anticancer and antioxidant activity [12]. Moreover, the toxic effects of this new compound have not yet been investigated. However, the toxic effects of exposure to these two xenobiotics remain distant from clear, and most studies have been performed on each contaminant separately. The need to recognize the eco-toxicological impacts of these xenobiotics on aquatic organisms has become essential, and links between the effects of NPs and phosphonates should be investigated.

Recently, biomarkers and chemical approaches have been used to evaluate the toxicological effects of xenobiotics on bivalves as filter-feeding organisms. It is well documented that bivalves have been utilized as indicators due to their tolerance against pollutants, having a central ecological position [13], and representing the potential transport of NPs through food chains [14]. In particular, the clam *Ruditapes decussatus*, abundant in the Mediterranean Sea, represents a suitable model organism for determining the impacts of NPs and phosphonates.

NPs and phosphonates can penetrate organisms and generate reactive oxygen species (ROS), causing increases in antioxidant enzyme activities [15] and neurotoxic effects [16]. In addition, NPs and phosphonate could lead to LPO increases and protein and DNA damage [17], as well as decreases in lysosomal membrane stabilities and phagocytic activity [18].

Due to widespread NPs and phosphonate occurrence in aquatic ecosystems and their toxic effects on different organisms, it is necessary to understand the mechanism of their toxicity after individual or combined exposure and to identify potential organ-based biomarkers for assessing biological interactions. From this perspective, the present research is focused on the mechanistic explanation of ZnS and P toxicities (separately and in combination) in the marine bivalve *R. decussatus,* and it illustrates the potential biomarkers for screening and monitoring the interaction and toxicity of these chemicals in marine organisms. In addition, considering the important role of the gills as a first barrier against the uptake of contaminants and of the digestive glands as key organs in energetic metabolisms [19,20], toxicity and interaction were evaluated in these organs to obtain a more specific ecotoxicological database.

## 2. Results

### 2.1. ZnS NPs Characterization

**Figure 1:**
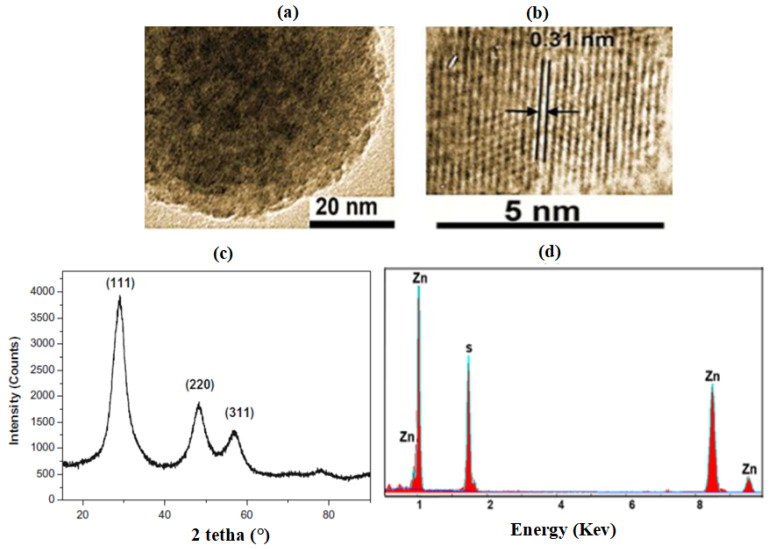
TEM images (**a**,**b**), DRX (**c**), and EDAX spectra (**d**) of ZnS nanoparticles.

The morphology of pure ZnS nanoparticles was determined by transmission electron microscopy. The TEM images in Figure 1a show that ZnS nanoparticles with a quasi-spherical shape are formed. The size of the prepared ZnS particles is about 5 nm. Lattice fringes can be clearly distinguished for a single ZnS nanocrystal (Figure 1b) as 0.31-nm interplane spacing of the (111) plane of ZnS in the cubic blende structure. Analysis by energy spectroscopy (EDAX) shows that the sample has only peaks of zinc and sulfur, thus confirming the high-purity ZnS nanoparticles (Figure 1c). The presence of peaks (<1 eV) is due to the copper grid used for the TEM/EDAX experiments.

### 2.2. Metal Content in the Clams

Table 1 shows that Fe, Cd, Pb, and Zn concentrations decreased after exposure to ZnS50 and P50 compounds separately in the whole soft tissue of *R. decussatus*. In contrast, metal content increased significantly (*p* < 0.05) in clams exposed to ZnS100. In addition, Cu concentrations increased significantly in clams with increased P concentrations, while Cd, Fe, Pb, and Zn decreased in P50- and P100-treated clams. Our results also showed an important increase in metal content in the whole soft tissue of clams treated with the mixture (M100).

### 2.3. Hydrogen Peroxide Levels

**Figure 2:**
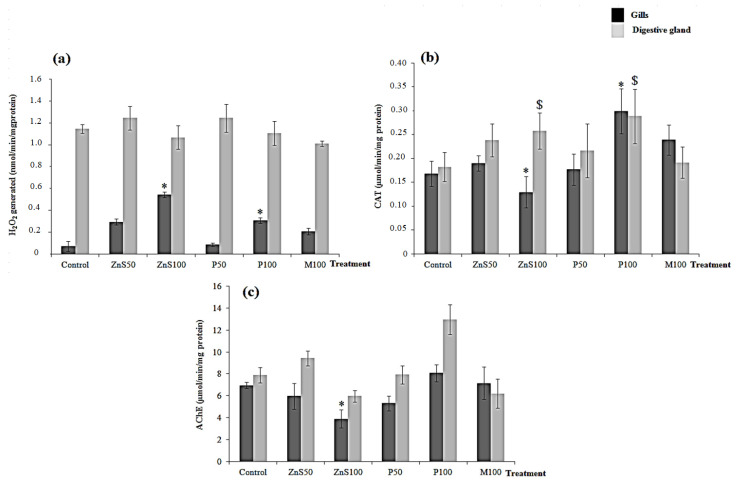
Antioxidant and neurotoxic effects of ZnS NPs and P: (**a**) hydrogen peroxide (H_2_O_2_) (**b**) CAT and (**c**) AChE in the gills and digestive glands of *Ruditapes decussatus* clams when exposed to increasing concentrations of ZnS NPs and P over 10 days. Values are the mean ± SD. (*) indicates significant differences (*p* < 0.05) and ($) indicates significant differences in digestive gland (*p* < 0.05).

The hydrogen peroxide levels were examined in both the digestive glands and gills of *R. decussatus* clam exposed to ZnS, P, and their combination (Figure 2a). No significant differences were observed in the digestive glands between control and treated clams. Moreover, ZnS50 and P50s showed no significant differences in H_2_O_2_ levels in gills compared to the control group. In the same organs, ZnS100 and P100 added separately showed significant increases in H_2_O_2_ levels by 76% and 62%, respectively, compared to the control group (*p* < 0.05). In contrast, no significant changes were observed in the gills and digestive glands of clams treated with M100 (*p* < 0.05).

### 2.4. Catalase Activity

No significant differences were observed in the gills and digestive glands of clams exposed to ZnS50 and P50 (Figure 2b). Significant increases in CAT activity were observed in the digestive glands of clams exposed to ZnS100 from 0.182 to 0.257 µmol/min/mg protein, but a significant decrease in this activity occurred in their gills. CAT activity was found to be stimulated after exposure to 100 µg/L per 3 g of compound from 0.168 to 0.2990 µmol/min/mg protein in gills and 0.182 to 0.288 in digestive glands (Figure 2b). In contrast, no significant changes in CAT activities occurred in the gills and digestive glands of clams exposed to the mixture of ZnS NPs and P compound (*p* < 0.05).

### 2.5. AChE Activity

No significant differences were observed after 10 days of exposure to ZnS50 and P50 in both gills and digestive glands (Figure 2c). In contrast, significant inhibition (*p* < 0.05) was detected in gills by 28.2% and digestive glands by 13.77% of specimens exposed to ZnS100. The exposure of clams to M100 did not change AChE activity in the gills and digestive glands compared to the control group (Figure 2c).

## 3. Discussion

### 3.1. Metal Content in Clams

The study of human impact on bivalves is of great significance from ecological, economic, and public health points of view. The results of the present study add more data about the toxicity caused by NPs and pharmaceutical compounds, particularly ZnS NPs, and phosphonates. These findings represent the first data on the in vivo effects of ZnS NPs and phosphate P added separately or in combination with metal variations and the enzymatic status of clams. The results demonstrate that exposure to ZnS NP suspensions induces significant changes in metal content in clams. *R. decussatus* accumulate Fe, Cu, and Zn in the total tissue. In agreement with our results, Vale et al. [21] reported an increase in metal concentrations in *Danio rerio* fish exposed to increased ZnO NP concentrations. These data suggest that metal accumulations are related to chemical and biochemical processes of detoxification of free radicals generated by NPs. It is also important to note that Cd, Fe, and Cu levels increased with increasing ZnS NPs concentrations in clam tissues. This outcome is probably due to the adsorption of these metals by ZnS NPs [21]. Eventually, ZnS NPs increased the bioavailability of these metals initially present in seawater, and this speciation allows the importation of metals into the cells [21].

Exposure of clams to phosphate P induced a decrease in most metal concentrations in the clams’ tissues. These results could be attributed to the high affinity of this compound against metals, as described by Aouani et al. [22], who suggested that the P=O moiety is responsible for the metal’s ion chelating effect.

Nevertheless, the opposite effect was demonstrated in our study, showing that P induced an additive effect on metal uptake in the presence of ZnS NPs. Comparable results were reported by Gagné et al. [23] in several aquatic species contaminated with a mixture of ZnO and pharmaceuticals. Alsop and Wood [24] observed an additive effect on Cu levels in zebrafish contaminated with Cu and fluoxetine.

### 3.2. Oxidative Stress and Modulating Effects

Once passed through the membrane barrier, NPs and pharmaceuticals produce reactive oxygen species (ROS), causing oxidative stress [21,25,26]. Our results revealed that the highest concentration of ZnS NPs induces overproduction of hydrogen peroxide in the gills. These results were confirmed by those in the literature [27]. Additionally, Asharani et al. [28] and Hussain et al. [29] showed that Ag NPs contribute to high ROS production by released ions [30]. This overproduction is related to the photocatalytic properties of ZnS NPs [31]. These data suggest that ROS overproduction is related to the photocatalytic aspect of ZnO NPs, and this disturbance could be the result of released Zn^2+^ in cells [32]. In the present study, P100 increased H_2_O_2_ levels only in gills, whereas no excessive production was observed with P50. This increase is related to the chemical nature of this phosphonate. According to Liendro et al. [33], organophosphorus compounds led to ROS production in *Oryzias latipes.* Similar effects were reported by Bebianno et al. [34] and Maranho et al. [35] in *R. philippinarum* and *R. decussatus* after exposure to a mixture of organophosphorus compounds. González et al. [36] and Cruz et al. [37] indicated that pharmaceutical products accumulated in *R. decussatus* clams, contributing to free radical generation.

Unlike metal uptake, exposure to a mixture of ZnS NPs and P induces an antagonistic effect against ROS production. This decrease is related to the ability of α-hydroxyphosphonate to neutralize excess hydrogen peroxide levels, as reported by Naidu et al. [38]. The addition of P opposes the toxic effects of nanoparticles by preventing ROS overproduction. Thus, both phosphorous and nitrile groups present in P mobilize and remove H_2_O_2_ from the organism [22]. In addition, Rao et al. [12] confirmed that phosphorous density has a crucial role in controlling oxygenated species affinity. It is also important to mention that metals react directly with ROS. However, chemical analysis by ICP shows that these two xenobiotics stimulate the non-enzymatic molecular defense system. Similarly, copper, zinc, and iron concentrations increase as a result of exposure to catalyst H_2_O_2_ levels, as reported by Fenton [39]. Zinc and copper ions are also catalysts of this radical, according to Mohanty and Samanta [40]. Similarly, iron is likely to induce a reduction in intracellular H_2_O_2,_ according to [36].

Antioxidant enzymes, such as CAT, are involved in protecting organisms from the harmful effects of H_2_O_2_ generated by xenobiotics [41]. Our results showed normal CAT activity in the gills and digestive glands after exposure to ZnS50. Similar results were also found in *Crassostrea gigas* oysters exposed to 50 μg/L of ZnO NPs [19] and *Scrobicularia plana* exposed to 10 μg/L of CuO NPs [26]. In contrast, stimulation of enzymatic defense mechanisms was demonstrated in *Scrobicularia plana* [20] and *E. complanata* exposed to a higher concentration of ZnO NPs [23]. It has been shown that CAT activity is much more important in the digestive glands than in other organs in bivalves exposed to a wide variety of nanoparticles [42]. This finding confirms the large difference in response between the gills and the digestive glands of specimens exposed to ZnS100. The same pattern of development has been reported in *E. complanata* exposed to ZnO NPs (2 mg/L) [23]. In addition, our results are in agreement with the studies of Ali et al. [43], who found that CAT activity in the digestive glands of *Lymnaea luteola* exposed to ZnO NPs increases with increasing concentration. CAT inhibition in clams treated with ZnS100 could be attributed to hydrogen peroxide overproduction. Gomes et al. [44] showed that CAT inhibition in *Mytilus galloprovincialis* is due to the interaction of Ag NPs with the thiol group of CAT. Moreover, Mohanty and Samanta [40] showed that CAT inhibition is directly related to the reaction between metal ions and the -SH group of this enzyme and indirectly with the superoxide anion [45].

CAT activity is also known to be strongly influenced by pharmaceuticals [37]. In the present study, the high concentration of P stimulated the antioxidant defense system in a concentration-dependent manner. Aquatic invertebrates are among the most sensitive aquatic species to pharmaceuticals. Martin-Díaz et al. [46] and Parolini et al. [47] showed that the antioxidant defense system’s activation against high levels of H_2_O_2_ in the digestive glands of *M*. *galloprovinciallis* and *Dreissena polymorpha* are dependently exposed to concentrations of pharmaceuticals. Our results showed a gradual increase in CAT activity in clams contaminated with two concentrations of P (P50 = 50 μg/L and P100 = μg/L).

CAT activity remains intact after exposure to the mixture (M100) of ZnS NPs and P. This result is related to the phosphonate compound P acting as an antagonist against ZnS NPs by a mechanism that has not yet been developed.

Aouani et al. [22] suggested that P possesses the ability to chelate DPPH radicals by donating an electron. Since the phosphorus group has an affinity for oxygen, it is also possible to have an affinity toward ROS, leading to a decrease in CAT activity. Our results prove that this compound can chelate free radicals, and then it can constitute a defense agent against the accumulation of NPs in the gills and digestive glands. The possible interactions are still unclear, and the mode of action is not well elucidated. In contrast, a study performed on *R. decussatus* showed that the exposure of this species to a mixture of metal ions and pharmaceuticals led to an increase in CAT activity in the digestive glands [48].

### 3.3. Neurotoxic Effects of ZnS NPs and P

AChE is the most commonly used biomarker of neurotoxicity in invertebrates [15], and this enzyme is inhibited by several neurotoxic agents [49]. The results of our study showed that ZnS NPs and P inhibit AChE activity in *Ruditapes decussates* at high concentrations without leading to muscle tetanization since no mortalities were observed during the experiment.

AChE inhibition is a concentration-dependent response since ZnS50 and P50 had no significant changes in clams, and the inhibition is observed in specimens treated with high concentrations (ZnS100 and P100). AChE activity was inhibited in *Hedist diversicolor* and *Nereis diversicolor* annelids exposed to increasing concentrations of ZnO [20]. Similarly, CuO NPs have been shown to contribute ta significant inhibition of AChE activity in *R. decussatus* after 5 days of exposure [34].

This inhibition of AChE is probably due to the adsorption of ZnS NPs to AChE active sites. This observation was confirmed by the hypothesis of [50], who showed AChE inactivation by Zn^2+^ ions. The denaturation of serine residues at the active site of AChE by Zn^2 +^ ions may also be the cause of AChE inhibition and the accumulation of acetylcholine in the synaptic space [51]. The theory of a modification of the electrostatic field of AChE by ions was also suggested by Radic et al. [52].

AChE inhibition is strongly correlated with a crucial increase in hydrogen peroxide after exposure to ZnS NP100 in the gills and digestive glands and to P100 in the gills. This result is in agreement with Schallreuter et al. [53], who explained that AChE activity becomes inactive due to the high production of H_2_O_2_. In addition, dependence was demonstrated by Antonio et al. [54], who discovered a positive correlation between the increase in CAT activity and the inhibition of AChE activity in aquatic species. In addition, Buffet et al. [26] showed that copper, zinc, and silver nano-oxides did not cause any changes in AChE activity in *Scrobicularia plana* clams treated with 100 μg/L. This finding proves that AChE activity is very variable between species. Pan et al. [9] also showed stimulation of AChE activity in *S. plana* contaminated with 100 mg/L of AuNPs and that this increase was associated with a compensatory response.

AChE is highly sensitive to pharmaceutical product variation [35]. Twenty percent or more of a decrease in AChE activity following exposure to any contaminant is considered a consequence of neurotoxicity caused by this pollutant [55]. Based on this theory, we can conclude from our results that P at a relatively high concentration has a neurotoxic effect on *R. decussatus* clams.

Similar neurotoxic effects have been observed in *R. decussatus* exposed to OMTOS (2-(4-methoxyphenyl)-5,6-trimethylene-4H-1,3,2-oxathiaphosphorin-2-sulfide) [56]. The inhibition of AChE activity was also demonstrated by Mattson et al. [57], who showed that a pharmaceutical (neguvon, an antiparasitic) inhibited this activity in salmonids and fish.

Brewer et al. [58], Sandahl et al. [59], and Sturve et al. [60] also showed inhibition of AChE by organophosphorus compounds. These authors suggested that the decrease in AChE activity in *Terapon jarbua* fish is strongly correlated with the bioaccumulation of organophosphorus products. In addition, Nunes et al. [61] found that, in the crustacean *Artemia parthenogenetica*, exposure to pharmaceutical substances, such as diazepam, inhibits AChE activity. Milan et al. [62] reported that inhibition of AChE activity is organo-dependent in *R. philippinarum* clams exposed to pharmaceuticals (100 μg/L) with greater inhibition in gills than in other organs, while the most recent studies showed that, for the same species contaminated with 50 μg/L of pharmaceutical product (antibiotics and antidepressants), AChE inhibition is more important in the digestive glands [63]. These authors have linked the neurotoxic effects of these pharmaceuticals with their modes of action. These products are known for their ability to inhibit AChE activity [64]. This relationship explains the inhibition of AChE by P, known for its antibacterial activity according to Pokalwar et al. [65] and Reddy et al. [66].

Contrary to the neurotoxic effects of P in the gills, significant stimulation of AChE activity in the digestive glands was observed after the administration of P100. This increase in AChE activity can be explained by an overcompensation of the gene codes for this enzyme and a reduction in the gene code for the acetylcholine vesicle transporter to decrease neuronal hyperexcitation [67]. The increased concentrations of acetylcholine are at the origin of continuous nerve transmission. The regulation of the expression of these genes makes it possible to decrease the availability and/or increase the hydrolysis of the neurotransmitter at the level of the synaptic space. In addition, this increase may be the consequence of the production of new forms of AChE, which trap inhibitors of this enzyme (AChE-R) and which hydrolyzes acetylcholine (AChE -S) [68].

Aguirre-Martinez et al. [69] showed an activation of the cholinergic system in clam *R. philippinarum* exposed to several families of pharmaceutical products. These authors have shown that exposure to an antibacterial drug (Novobiocin = 0.1 to 50 μg/L) and another anti-inflammatory product (Ibuprofen = 5 to 50 μg/L) is likely to induce a progressive increase in AChE activity in the digestive gland. Similarly, AChE stimulation in clam was also detected following contamination with a low concentration of caffeine (0.1 μg/L). Aguirre-Martinez et al. [69] pointed out that this stimulation is closely linked to the level of contamination and is probably contaminant-dependent. This was confirmed by Aguirre-Martinez et al. [69], which corroborated the hypothesis of a significant increase in AChE as a transient effect of the drug, suggesting that clams attenuate neurotoxic effects by increasing AChE activity in the digestive gland. Zhang and Greenberg, [70] and Oliveira et al. [63] also indicated that the cholinergic system activation offers the advantage of being a powerful indicator of neurotoxicity in clams exposed to a pharmaceutical product. The authors suggest that this increase may be a coping mechanism but not a defense against stress [71].

The mixture of ZnS NPs and P at similar concentrations showed an antagonist AChE response. This outcome would suggest that P possesses the ability to activate the cholinergic system in the presence of NPs.

## 4. Materials and Methods

### 4.1. ZnS NPs and Diethyl (3-cyano-1-hydroxy-2-methyl-1-phenyl propyl) phosphonate Synthesis

To synthesize the ZnS NPs, zinc acetate dihydrates [Zn (OAc) _2_. 2 H_2_O) Aldrich, AR grade] (1.15 g) and thiourea (0.47 g) were dissolved in 25 mL of 1,3-propanediol, and the mixture was heated to 190 °C and kept at this temperature for 2 h under continuous magnetic stirring. At the end of the reaction, the precipitate was centrifuged, washed several times with ethanol, and then dried in a vacuum at 60 °C for 12 h to yield a white, dry ZnS powder. The crystalline structure of the obtained powder was characterized by X-ray diffraction (XRD) (an INEL diffractometer with a copper anticathode (λ = 1.54060 Å)). High-resolution TEM (Philips Tecnai F-20 SACTEM operating at 200 Kv) images provided further insight into the structural information of the ZnS NPs.

**Scheme 1:**
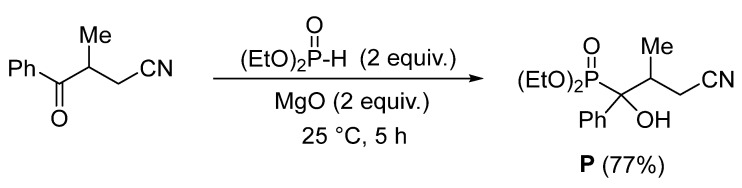
Synthesis of phosphonate P.

Diethyl (3-cyano-1-hydroxy-2-methyl-1-phenyl propyl)phosphonate (P) was synthesized in 77% yield, according to the procedure reported by Aouani et al. [22], which involves the reaction of 3-methyl-4-oxo-4-phenyl-butane nitrile with diethylphosphite at room temperature for 5 h in the presence of magnesium oxide as solid support (Scheme 1).

Experimental procedure: Diethylphosphite (0.02 mol) was added to the ketonitrile (0.01 mol), and the mixture was heated at 60 °C and stirred until the ketonitrile was dissolved. Then, magnesium oxide (0.02 mol) was added, and the mixture was stirred at 25 °C for 5 h. The magnesium oxide was filtered and washed with dichloromethane (3 × 10 mL). The filtrate was extracted with water (2 × 30 mL). The organic phase was dried over Na_2_SO_4_ and concentrated under a vacuum. The obtained residue was chromatographed on a silica gel column using a mixture of ether and hexane (8:2) as an eluent.

The physical and spectral data for compound P were: brown solid; mp 182-184 °C; ^31^P NMR (121.5 MHz, CDCl_3_): δ(ppm) = 21.6 (min), 22.0 (maj); maj/min: 61/39; ^1^H NMR (300.1 MHz, CDCl_3_): δ (ppm) = 0.94–1.46 (m, 6H, CH_3_-CH_2_-O); 2.16 (d, 3H, ^3^J_HH_ = 9.0 Hz, CH_3_-CH, min); 2.20 (d, 3H, ^3^J_HH_ = 9.0 Hz, CH_3_-CH, maj); 2.49–2.78 (m, 2H, CH-CH_2_-CN); 3.50–3.82 (m, 1H, CH-CH_2_-CN); 3.98–4.22 (m, 4H, CH_3_-CH_2_-O); 5.22 (br s, 1H, OH, min); 5.52 (br s, 1H, OH, maj); 7.12–7.62 (m, 5H, arom-H); ^13^C NMR (75.5 MHz, CDCl_3_): δ (ppm) = 13.7 (d, ^3^J_CP_ = 8.2 Hz, CH-CH_2_-CN); 16.2 (d, ^3^J_CP_ = 6.0 Hz, CH_3_-CH_2_-O); 16.4 (d, ^3^J_CP_ = 6.0 Hz, CH_3_-CH_2_-O); 19.8 (s, CH_3_-CH, min); 20.2 (s, CH_3_-CH, maj); 37.7 (d, ^2^J_CP_ = 7.0 Hz, CH-CH_2_-CN); 61.1 (d, ^2^J_CP_ = 6.0 Hz, CH_3_-CH_2_-O); 61.4 (d, ^2^J_CP_ = 6.0 Hz, CH_3_-CH_2_-O); 75,8 (d, ^1^J_CP_ = 160.5 Hz, P-C-OH, maj); 79.1 (d, ^1^J_CP_ = 161.8 Hz, P-C-OH, min); 118.5 (s, CN, maj); 119.5 (s, CN, min); phenyl carbons: δ (ppm) = 125.5, 125.7, 125.9, 127.4, 127.7, 127.9, 128.1, 128.2, 128.3, 128.4, 128.6, 128.9, 132.9, 133.7; IR (neat): ν_P=O_ = 1256 cm^−1^, ν_CN_ = 2246 cm^−1^, ν_OH_ = 3483 cm^−1^; EI-HRMS: calculated for C_15_H_22_NO_4_P: 311.1286 (M^+^), found: 311.1282.

### 4.2. In Vivo Exposure

Clams (*R. decussatus*) of between 4.22 and 3.25 cm in shell length were collected in the Bizerte lagoon, Northern Tunisia. Acclimation occurred in 2-L glass tanks for 1 week before starting the exposure. The following experimental treatments were established in triplicate tanks. Clams were divided into groups of 5 per tank and exposed at the same time to ZnS NPs, P, or a combination of the 2 compounds (M100) for 10 days. Two concentrations of 50 and 100 µg L^−1^ were considered for ZnS NPs and diethyl (3-cyano-1-hydroxy-1-phenyl-2-methyl propyl) phosphate or P. The exposure treatments were labeled as follows: ZnS50 and ZnS100 for dissolved 50 and 100 µg L^−1^ of ZnS, P50; and P100 for dissolved 50 and 100 µg L^−1^ of phosphonate and M100 for dissolved ZnS100 and P100 mixture.

A control series without ZnS NPs and P was run in parallel. Aeration at the bottom of the tank was used to minimize agglomeration and subsequent sedimentation of the contaminants, and the seawater was changed every 48 h. For the duration of the experiment (10 days), the clams were fed, with the seawater changed every 48 h. Test animals were checked daily. No mortality was observed, and all animals were seen to be feeding normally.

### 4.3. Metal Analysis

Heavy metals and electrolytes present in the clams were determined using an Optima 7300 DV optical emission spectrometer. After 10 days of exposure, the clams were dissected, and the whole tissue was weighed. The whole tissue was then placed in the oven at 105 °C for 30 min to be dried. The samples are mineralized separately by a mixture of HNO_3_ 5% (3 mL), H_2_O_2_ (3 mL), and H_2_O (1 mL). The digestion was continued in a microwave for 20 min at 720 W. The samples were diluted with 50 mL of ultrapure water [72]. The solution was filtered to eliminate the organic particles that remained in the suspension and entrainer interferences during measurement. Finally, the samples were analyzed by ICP-AES using reference material of mussel tissue (NIST 2976) [73]. The detection limits for Cd, Zn, Fe, Cu, and Pb were in the range of 0.006–25 mg/L. The results are expressed in g kg-1 wet weight. Values reported were corrected for background levels determined in blank sterile filtered seawater.

### 4.4. Biochemical Analysis

After 10 days of exposure, the digestive glands and gills of each individual were dissected on ice. Protein extractions were performed and homogenized by a polytron homogenizer in 10 mMTris/HCl with a pH of 7.2, containing 500 mM sucrose, 1 mM EDTA and 1 mM PMSF; supernatants were collected by centrifugation at 9000× *g* (4 °C for 30 min). Protein concentrations were quantified according to Bradford’s [74] method based on a colorimetric reaction using bovine serum albumin (BSA). Hydrogen peroxide (H_2_O_2_) levels were measured following the method of Wolff [75]: a volume of 0.1 mL of the supernatant was added to 900 mL of FOX1 reagent (100 mxylenol orange, 100 mM sorbitol, 250 mM ammonium ferrous sulfate, and 25 mM H_2_SO_4_), vortexed, and incubated at room temperature for 30 min. The sample was then centrifuged at low speed for 3 min, and the absorbance of the supernatant was read at 560 nm. CAT activity was measured by the decrease in absorbance at 240 nm due to H_2_O_2_ consumption according to the method of Aebi [76]. The reaction volume and reaction time were 1 mL and 1 min, respectively. The reaction solution contained 80 mM phosphate buffer with a pH of 6.5 and 50 mM H_2_O_2_ [77]. Specific CAT activities are reported as µmol/min/mg protein.

AChE activity was determined using the method of Ellman et al. [78]. Measurements of AChE activity were initiated by the addition of acetylthiocholine, and then absorbance was read at 412 nm. Results were expressed in µmol/min/mg of the hydrolyzed substrate relative to the total protein content.

### 4.5. Data Analysis

Metal content, H_2_O_2_ levels, and enzymatic activities were compared among samples using Statistica software, version 8.0. After testing ANOVA assumptions, statistical significance was evaluated through one-way ANOVA. Tukey’s HSD test allowed pairwise comparisons between experimental conditions, and a significant difference was considered at *p* < 0.05.

## 5. Conclusions

The toxicity of phosphonates, specifically diethyl (3-cyano-1-hydroxy-2-methyl-1-phenyl propyl) phosphonate (P) and ZnS NPs, in marine clams is highlighted in the present study. The study revealed that phosphonate had both additive and antagonistic toxic effects when present with ZnS NPs. Consequently, exposure to these pollutants disrupted the metal content and oxidant/antioxidant balance in the clams’ gills and digestive glands, leading to an increase in CAT activity, H_2_O_2_ overproduction, and neurotoxic effects. The results suggest that phosphonate could be utilized as a new product to control NP toxicity in aquatic environments. To verify the findings of this initial study, additional tests examining the toxicity of ZnS NPs and P at various concentrations are necessary.

## Figures and Tables

**Table 1:** Metal content in the whole soft tissues of control and treated clams (g/kg dw).

	Cd	Cu	Fe	Pb	Zn
Control	0.069	0.025	0.750	0.28	2.098
ZnS50	0.001	0.2	0.22	0.02	0.690
ZnS100	0.130	0.098	1.25	0.155	3.968
P50	0.001	0.290	0.320	0.090	0.710
P100	0.001	0.250	0.470	0.090	1.090
M100	0.224	0.545	2.750	0	13.112

## Data Availability

All the data in the article are available from the corresponding author upon reasonable request.

## Sample Availability

Samples of all the compounds are available from the authors.
